# Description of the Wild Strain *Rhizobium rosettiformans* DSM26376, Reclassified under *Peteryoungia rosettiformans* comb.nov., for Producing Glucuronan

**DOI:** 10.3390/polym15092177

**Published:** 2023-05-03

**Authors:** Gwendoline Christophe, Xiaoyang Hou, Emmanuel Petit, Mounir Traikia, Didier Le Cerf, Christophe Rihouey, Christine Gardarin, Cédric Delattre, Philippe Michaud, Guillaume Pierre, Pascal Dubessay

**Affiliations:** 1 Clermont Auvergne INP, CNRS, Institut Pascal, Université Clermont Auvergne, F-63000 Clermont-Ferrand, France; 2 UMRT INRAe 1158 BioEcoAgro, Laboratoire BIOPI, Institut Universitaire et Technologique, Université de Picardie Jules Verne, F-80025 Amiens, France; 3 CNRS, ICCF, Université Clermont Auvergne, F-63000 Clermont-Ferrand, France; 4 Polymères Biopolymères Surfaces, Normandie Université, UNIROUEN, INSA Rouen, CNRS, UMR6270, F-76821 Mont Saint-Aignan, France; 5 Institut Universitaire de France (IUF), 1 rue Descartes, F-75005 Paris, France

**Keywords:** *Peteryoungia rosettiformans*, rhizobium, exopolysaccharide, glucuronan, glucuronan lyase

## Abstract

Glucuronan is a polysaccharide composed of β-(1,4)-linked d-glucuronic acids having intrinsic properties and biological activities recoverable in many fields of application. Currently, the description of *Sinorhyzobium meliloti* M5N1CS mutant bacterial strain as the sole source of glucuronan makes it relevant to the exploration of new microorganisms producing glucuronan. In this study, the *Peteryoungia rosettifformans* strain (Rhizobia), was identified as a wild producer of an exopolysaccharide (RhrBR46) related to glucuronan. Structural and biochemical features, using colorimetric assays, Fourier infrared spectroscopy, nuclear magnetic resonance, high pressure size exclusion chromatography coupled to multi-angle light laser scattering, and enzymatic assays allowed the characterization of a polyglucuronic acid, having a molecular mass ($\bar{M_{w}}$) of 1.85 × 10^5^ Da, and being partially *O*-acetylated at *C*-2 and/or *C*-3 positions. The concentration of Mg^2+^ ions in the cultivation medium has been shown to impact the structure of RhrBR46, by reducing drastically its $\bar{M_{w}}$ (73%) and increasing its DA (10%). Comparative structural analyses between RhrBR46 and the glucuronan from *Sinorhyzobium meliloti* M5N1CS strain revealed differences in terms of molecular weight, degree of acetylation (DA), and the distribution of acetylation pattern. These structural divergences of RhrBR46 might contribute to singular properties or biological activities of RhrBR46, offering new perspectives of application.

## 1. Introduction

Glucuronan is a linear homopolymer composed of β-(1,4)-linked d-glucuronic acids, with variable degrees of *O*-acetyl substitution at *C*-2 and/or *C*-3 positions. Overall, glucuronan is considered a low abundant polysaccharide, compared with other existing polyuronates, and is mainly found as a structural component of the cell walls of some microorganisms. Polyglucuronic acids can be alternatively synthesized in a chemical way using 2,2,6,6-tetramethylpiperidine-1-oxyl radical (TEMPO) allowing regioselective oxidation of natural glucans [1,2]. TEMPO-mediated oxidation is notably extensively used to oxidize the cellulose (C6 primary hydroxyl into carboxylate group) into a water-soluble β-(1,4)-d-polyglucuronic acid (cellouronic acid) considered a mimetic of glucuronan [3,4].

The natural sources of polyglucuronic acids are limited to a few microorganisms, for which β-(1,4)-linked polyglucuronate or related glucuronan-rich patterns in natural polymers have been identified as components of exopolysaccharides (EPS) and/or cell wall structures [5]. β-(1,4)-d-polyglucuronic acids of low molecular weights have been isolated and characterized from the cell wall of fungi such as *Mucor rouxii* (mucoric acid, polymer II) [6,7] or *Trichosporon cutaneum* [8]. Glucuronan has also been found as part of the heterogeneous polysaccharide structure of some marine green algae cell walls [9]. Bacteria constitute the main and most documented source of natural glucuronan, including both oligo- and poly-glucuronic acids associated with divergent structural features, rheological behaviors, and biological properties [10,11,12]. Two alternative glucuronan, an α-(1,4)-oligoglucuronic acid and an alternating α- and β-(1,4)-glucuronan can, respectively, be produced by *Gluconacetobacter hansenii* PJK (KCTC 10505BP) [13] and an alkalophilic Bacillus strain *C-125* [14]. A mutant bacterial strain *Sinorhizobium meliloti* M5N1CS (NCIMB 40472), generated by chemical mutagenesis of *Sinorhizobium meliloti* M5N1 using N-methyl-N’-nitro-N-nitrosoguanidine, acquired the ability to produce an extracellular glucuronan, described as a homopolymer of β-(1-4) linked d-glucuronic acids, with an acetylation degree of 50% covering *C*-2 and/or *C*-3 positions [15,16,17]. The Mg^2+^ ions impact the structural features of this glucuronan, by reducing its size and increasing its degree of acetylation [17]. The metabolic pathways shifted by the mutagenesis and leading to the production of glucuronan rather than succinoglycan remains unknown.

Owing to their intrinsic properties and biological activities, β-(1,4)-d-poly- and oligo-glucuronic acids are of first interest in many fields, ranging from food, pharmaceuticals, farming, and cosmetics to water purification. Regarding the literature, several patents described their properties as gelling, thickening, stabilizing, hydrating, and chelating agents [9]. The activity of poly- or oligo-glucuronic acids in the immunostimulating of human monocyte and cytokines induction [18], the stimulation of skin layers elasticity induced by acetylated oligo-glucuronan [19], and its potential use as an elicitor agent to enhance plant natural defenses [20] have also been described.

The exploitation of β-(1,4)-d-poly- and oligo-glucuronic acids in many fields makes it relevant to explore new microorganisms producing glucuronan. Although bacteria from the Rhizobiaceae family are claimed to be exopolysaccharide producers [21], only a few species have been investigated for their capacity to produce glucuronan. In this work, we described the identification of a *Peteryoungia rosettiformans* strain as a producer of a medium-weight (1.85 × 10^5^ Da) poly-glucuronic acid. This strain, initially referenced as *Rhizobium rosettiformans* DSM26376 (DSMZ-German Collection of Microorganisms and Cell cultures GmbH) and previously isolated from a hexachlorocyclohexane dump site (Lucknow, India) [22], was recently reclassified under a novel genus *Peteryoungia* based on phylogenomics studies of Rhizobiaceae [23]. The structural features of the EPS produced by *P. rosettiformans* have been fully studied allowing us to describe the first-time production of glucuronan by a wild strain belonging to Rhizobiaceae.

## 2. Materials and Methods

### 2.1. Biological Materials and Chemicals

The *Rhizobium rosettiformans* DSM26376, recently reclassified as *Peteryoungia rosettiformans* [23] strain, was purchased from DSMZ-German Collection of Microorganisms and Cell Cultures GmbH (Leibniz Institute, Leibniz, Germany). The glucuronan used as reference (SmPGU) was a gift from Pr. Petit (Laboratoire BIOPI, University of Picardie, France). All chemicals were purchased from Sigma-Aldrich^®^ (Saint-Quentin-Fallavier, France) and were of analytical grade.

### 2.2. Peteryougia rosettiformans Strain Cultivation

The *P. rosettiformans* (*Rhizobium rosettiformans* DSM26376) strain was cultivated and maintained at 30 °C under agitation on a rotary shaker (130 rpm) (Shaking Incubator Cooling NB-250LF, N-BIOTEK^®^, Pyeongcheon-ro, Korea) in Rhizobium Complete (RC) medium (Yeast Extract 1 g∙L^−1^, K_2_HPO_4_ 1 g∙L^−1^, MgSO_4_∙7H_2_O 0.2 g∙L^−1^, pH 7.2) supplemented with sucrose 1% (wt/vol) (RCS medium) [24].

### 2.3. EPS Production in Bioreactor

Concerning the EPS production, a culture of 50 mL (RCS medium), inoculated with *P. rosettiformans* was carried out overnight at 30 °C under agitation (130 rpm). Ten milliliters of the overnight culture were used to inoculate 300 mL of RCS medium in a 500 mL Erlenmeyer, maintained at 30 °C (130 rpm). The bacterial growth was monitored by measuring optical density at 600 nm (OD_600_) (V-630 spectrophotometer, Jasco^®^, Lisses, France). When OD_600_ reached 0.4–0.5, 300 mL preculture was transferred into a 5 L bioreactor (MiniPro-Lab, Global Process Concept GPC^®^, Perigny, France), containing 2.7 L of RCS medium. The pH in the reactor was maintained at 7.2 using 1 M KOH. The partial oxygen pressure (pO_2_) was stabilized at 80% during the first 24 h, corresponding to the exponential growth, and at 40% until the end of the fermentation process (~70 h). During the process, several samples were collected to monitor both the bacterial growth and the kinetics of the EPS production. The bacterial growth was estimated by measuring both the optical density (OD_600_) and dry weight of biomass. The kinetics of the EPS production was investigated by total sugars assay using the Dubois method [25] (see details in Section 2.5), from 50 mL of the fermenter culture after removal of bacteria by centrifugation (10,000× *g* for 30 min) (Heraeus Biofuge PRIMO Rcentrifuge, Thermo Scientific^®^, Illkirch-Graffenstaden, France) and three successive precipitations of the EPS with three volumes of isopropanol, and lyophilization (Heto PowerDry PL6000 Freeze Dryer, Thermo Scientific^®^).

Based on previous studies [17], a second culture in the bioreactor was performed to investigate the effect of Mg^2+^ on EPS production. The experimental parameters and conditions were as described above, except for the use of a higher concentration of MgSO_4_∙7H_2_O (1.2 g∙L^−1^) in RCS^++^ medium.

### 2.4. EPS Isolation and Purification

After 70 h of fermentation, the culture was collected, and the bacteria were removed by centrifugation (10,000× *g*, 30 min, 4 °C) (Avanti^®^ J-E Centrifuge, Beckman Coulter). The EPS was precipitated by adding three volumes of cold isopropanol to the supernatant and placed at −20 °C for 24 h. The solution was then centrifuged (10,000× *g*, 30 min, 4 °C), the supernatant was discarded, and the pellet was re-suspended in 100 mL of ultra-pure water. This step of alcoholic precipitation was repeated three times to eliminate a large part of the salts. The resulting fraction was desalted and concentrated by tangential filtration using a cassette system (Vivaflow 200, Sartorius, Göttingen, Germany) with a cutoff threshold of 100 kDa, and the conductivity was monitored using Session + EC7 conductivity meter apparatus (Hach^®^, Lognes, France). The EPS solution was then lyophilized and stored at ambient temperature. The final lyophilized products obtained from RCS medium and RCS^++^ medium fermentations were, respectively, named RhrBR46 and RhrBR46-Mg.

### 2.5. Chemical Compositions of RhrBR46 and RhrBR46-Mg

The global compositions of RhrBR46 and RhrBR46-Mg were carried out by colorimetric assays. Total carbohydrate was quantified by the procedure of Dubois [25] using glucose as the standard. Neutral sugars and uronic acid content were, respectively, determined by the sulfuric resorcinol assay using glucose as reference [26] and the *m*-hydroxybiphenyl method [27] using glucuronic acid as a standard. The correction method proposed by Montreuil [28] was applied to calculate neutral and uronic acid sugar concentrations. Protein content was quantified by the micro-Bradford assay using bovine serum albumin (BSA) as a standard [29]. Total phenolic compounds content was determined by the Folin-Ciocalteu procedure using gallic acid as a reference [30]. The conductivity (µS∙cm^−1^) was monitored using Session + EC7 conductivity meter apparatus (Hach^®^, Lognes, France), and the equivalent [NaCl] (g∙L^−1^) was determined based on a standard curve.

### 2.6. Determination of Molecular Characteristics

#### 2.6.1. HPSEC-RID Analyses

As a first approach, the molecular weights of both fractions were estimated by high-pressure size exclusion chromatography (HPSEC) (HPLC 1100 series, Agilent, Palo Alto, CA, USA) coupled with a refractive index detector (RIDG1362, Agilent), using pullulan standards (1.3–800 kDa). Two sets of two columns (TSKgel PWXL Type Guard column 5000/3000 or 3000/2500, Tosoh Bioscience GMBH, Stuttgart, Germany) were eluted with sodium nitrate (NaNO_3_, 0.1 M) at 50 °C and 1 mL∙min^−1^. Both RhrBR46 and RhrBR46-Mg were solubilized for 24 h under stirring (350 rpm) at room temperature in NaNO_3_ (0.1 M) before their injections (20 µL). The number (M_n_) and weight (M_w_) average molecular weights and the polydispersity index (Đ) were calculated using the following Equations (1)–(3):

(1) $$M_{n}=\frac{\sum N_{i}M_{i}}{\sum N_{i}}$$

(2) $$M_{w}=\frac{\sum N_{i}M_{i}{}^{2}}{\sum N_{i}M_{i}}$$

(3) $$Đ=\frac{M_{w}}{M_{n}}$$

where M_i_ and N_i_ are the molecular weight and number of moles of polymer species, respectively.

#### 2.6.2. HPSEC-MALS Analyses

Getting further macromolecular details, RhrBR46 and RhrBR46-Mg were analyzed by HPSEC comprising on line detectors including a multi-angle laser light scattering (MALS) filled with a He-Ne laser at 690 nm and a K5 cell (50 µL) (HELEOSII, Wyatt Technology Corp., Goleta, CA, USA) equipped with 18 measuring diodes and calibrated with Toluene and BSA, and a differential refractive index (DRI) (RID10 A Shimadzu, Kyoto, Japan). Columns (OHPAK 804 and 806 HQ columns (Shodex, Agilent Technologies, Les Ulis, France)) were eluted with LiNO_3_ (0.1 M) at 0.5 mL∙min^−1^. RhrBR46 and RhrBR46-Mg were solubilized at 1 g∙L^−1^ in LiNO_3_ (0.1 M) for 72 h at room temperature under stirring (200 rpm), filtered (0.45 µm) and then injected through a 500 µL full loop. The collected data were analyzed using Astra 6.17.16 software, a dn/dc value of 0.150 and 1st-order Zimm fits.

#### 2.6.3. FT-IR Analysis

Fourier-Transform Infrared (FT-IR) analyses were performed using a VERTEX 70 FT-IR instrument. Samples were dispersed on ATR A225 diamante. The FT-IR spectra were recorded at ambient temperature (referenced against air) in the wavelength range of 5000–400 cm^−1^, using a resolution of 4 cm^−1^ (50 scans). Spectra were analyzed with OPUS 7.2 software.

#### 2.6.4. NMR Spectroscopy

One hundred mg of RhrBR46 or RhrBR46-Mg were dissolved in 1 mL of D_2_O (99.9% D) and freeze-dried (three times) to substitute deuterium exchangeable protons. Before NMR spectroscopy, both fractions were dissolved in D_2_O (50 g∙L^−1^) for 24 h at room temperature under stirring (350 rpm) and then analyzed at 80 °C using a 400 MHz Bruker Avance Spectrometer (Germany) equipped with a BBFO probe. The NMR experiments were applied with a spectral width of 3000 Hz with the following acquisition parameters: (i) for ^1^H experiments, recovery = 5 s (for a complete return after a 90° pulse), number of scans > 60, acquisition mode = 2 s, pulse 90° = 8 µs; (ii) for ^13^C experiments, recovery = 2 s, number of scans > 16,000, acquisition mode = 0.34 s, pulse = 7 µs, accumulation > 10 h.

#### 2.6.5. Determination of the Degree of Acetylation

The degree of acetylation (DA) was measured using the results from both FTIR and NMR ^1^H. Briefly, three methods were used for determining the DA (%) by FTIR by calculating various ratios of areas after deconvolution (Levenberg-Marquardt) [31,32], using Equations (4)–(6):

(4) $$\mathrm{DA}\;\left(\%\right)=\frac{A_{1725}}{A_{1045}}$$

(5) $$\mathrm{DA}\;\left(\%\right)=\frac{A_{1725}}{A_{1598}}$$

(6) $$\mathrm{DA}\;\left(\%\right)=\frac{A_{1725}}{\left(A_{1725}-A_{1598}\right)}$$

Two methods were used for determining the DA (%) and DAc (acetate number) by NMR:

- as the average number of acetates per glucuronic acid residue. It varies between 0 and 2, and expressed as a percentage, it varies between 0 and 200%. The integration of the proton resonances of the glucuronic acid units (4.3–5.8 ppm), and acetate resonance region (2.3–2.6 ppm) allows the calculation of DA (%) (Equations (7) and (8));
  (7) $$\mathrm{DAc}\;\left(\mathrm{per}\;\mathrm{residue}\right)=\frac{\mathrm{area}\;\mathrm{of}\;\mathrm{acetate}\;\mathrm{peaks}}{3\times \;\mathrm{area}\;\mathrm{of}\;\mathrm{proton}}$$
- as the average number of substituents per disaccharide unit of glucuronan. It is determined as being the average number of substituents per disaccharide unit of glucuronan. The integration of the resonances of the protons of the glucuronan corresponding to the peaks H, I, J, and K makes it possible to calculate the DAc. The area of a proton is determined by the sum of the areas of the protons H2, H3, H4, and H5 (H peaks) divided by 5 for a disaccharide unit. An acetyl group has three protons, therefore, the area of a proton of an acetyl group is the sum of the areas of the peaks I, J, and K divided by three (Equation (8)).
  (8) $$\mathrm{DAc}\;\left(\mathrm{per}\;\mathrm{disccharide}\right)=\frac{\frac{\sum \mathrm{areas}\;\mathrm{of}\;\mathrm{peaks}\;I,\;J,\;K}{3}}{\frac{\sum \mathrm{areas}\;\mathrm{of}\;\mathrm{peaks}\;H\;}{5}}$$

Finally, the distribution in acetates 2-*O*-Ac, 3-*O*-Ac, and 2,3-di-*O*-Ac was obtained by NMR ^1^H by the specific integration of the signal I with respect to the sum of the signals I, J, and K for the acetates in 2. The proportion of the acetates in positions 2 and 3 on the same residue was given by the integration of the signal K. The number of acetates in position 3 was obtained by difference [16].

#### 2.6.6. Enzymatic Degradability of RhRBR46 and RhrBR46-Mg by a Glucuronan Lyase (TrGL)

The enzymatic assays were performed using the recombinant glucuronan lyase from *Trichoderma reesei* (TrGL) produced in *Pichia pastoris* as described previously [33]. The glucuronan lyase activity was spectrophotometrically quantified measuring for 5 min the increase of A_235_ related to unsaturated products. Standard reactions were performed using 2 µg of the purified enzyme, at 25 °C in 50 mM potassium acetate buffer (pH 5.5) with solubilized substrates (2 g∙L^−1^), including RhrBR46, RhrBR46-Mg, and their deacetylated forms, as well as glucuronan from *S. meliloti* (SmPGU) used as a reference. The deacetylated forms were obtained by treatment with 2 M KOH for 12 h at 50 °C (pH 12) as described previously [24].

One unit of glucuronan lyase activity (U) was defined as that corresponding to the production of 1 µmol of unsaturated products per minute. The molar extinction coefficient of ∆-(4,5)-unsaturated oligo-glucuronan (degree of polymerization of 3) was assumed to be 4931 M^−1^∙cm^−1^ [33].

The kinetic parameters such as the Michaelis constant (K_M_), the catalytic constant (K_cat_), and the catalytic efficiency (K_cat_/K_M_) were measured at 25 °C, using 0.25–2.5 g∙L^−1^ of substrates. The K_M_ values were determined using Lineweaver–Burk plots. All the assays were repeated in triplicate.

## 3. Results and Discussion

### 3.1. Kinetic of EPS Synthesis by P. rosettiformans in Bioreactor

**Figure 1:**
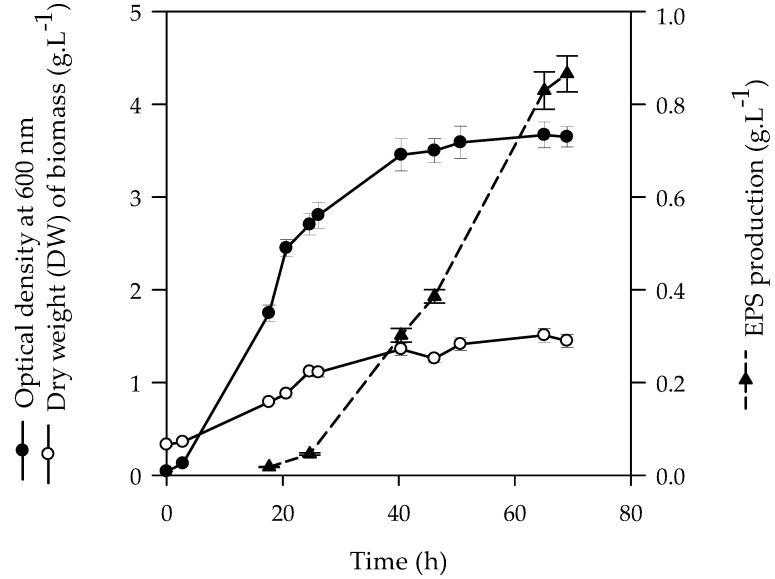
Biomass production (OD_600_ (●) and dry weights (○)) and EPS production (▲) during cultivation *of Peteryoungia rosettiformans* DSM26376.

Cultivation of *P. rosettiformans* DSM26376 in RC medium supplemented with sucrose 1% lead to an increase in its viscosity, suggesting the ability of this strain to produce EPS (named RhrBR46). The kinetic of RhrBR46 synthesis was monitored in a 3 L batch bioreactor according to pO_2_, pH, and stirring conditions described for *Sinorhizobium meliloti* M5N1CS [15]. The production of EPS was significantly detected in the medium at approximately 24 h, increased linearly from 24 h to 69 h, then finally reached a concentration of 0.8 g∙L^−1^ (Figure 1).

The kinetic of RhrBR46 synthesis by *P. rosettiformans* was like those generally described for other bacteria strains, for which EPS is produced as a secondary metabolite during the late log and stationary phases of growth, when there is a depletion of further nutritional sources (e.g., nitrogen) and favorable factors [34]. The EPS production (0.8 g∙L^−1^) by *P. rosettiformans* was relatively lower compared to other Rhizobia such as *R. tropici* (1.14 g∙L^−1^) or *R. meliloti* known for its ability to produce high levels of EPS (7.8 g∙L^−1^) [35,36]. Likewise, the EPS productivity in *P. rosettiformans* (0.27 g∙L^−1^/24 h) was lower than that of *S. meliloti* M5N1CS, (2 g∙L^−1^/24 h) [15].

The effect of Mg^2+^ and Mn^2+^ ions was proven to generally enhance EPS production as described for alginate biosynthesis and biofilm polysaccharides production by *Pseudomonas* sp. [37,38] or succinoglycan synthesis by *Rhizobium meliloti* SU47 [36]. In a second culture, the effect of Mg^2+^ on EPS production was investigated by cultivating *P. rosettiformans* in the same condition as RhrBR46 but with a higher Mg^2+^ concentration (1.2 g∙L^−1^ in RCS^++^ medium). The quantification of the EPS fraction (RhrBR46-Mg) collected at 69 h allowed us to estimate a production of 2.10 g∙L^−1^), which was 2.6 times higher compared to RhrBR46 extracted from the RC culture medium. Note that any antibacterial effect on the culture has been shown during the growth of the strain. The stimulating effect of Mg^2+^ on EPS production in *P. rosettiformans* could suggest the activation of enzymes involved in the synthesis of precursors for the EPS polymerization, as it has been previously reported for the alginate biosynthesis by *Pseudomonas aeruginosa* [37]. Moreover, it should be noted that Mg^2+^ may have, in some cases, a negative effect on EPS production, as observed for the glucuronan synthesis by *S. meliloti*, for which a decrease in EPS production was reported with increasing Mg^2+^ concentration [17]. The identification of a glucuronan lyase, produced in presence of Mg^2+^, led the authors to assign the reduction of EPS production to its degradation by the enzyme [24,39].

### 3.2. EPS Characterization

#### 3.2.1. Chemical Composition

The chemical compositions of RhrBR46 and RhrBR46-Mg are summarized in Table 1. Both fractions were mainly composed of carbohydrates, 78.22% and 65.17% for RhRBR46 and RhrBR46-Mg, respectively, indicating a relatively enriched-polysaccharide fraction.

No phenol compounds were detected, and the protein content was low (<0.40%). The efficiency of desalting steps during the ultrafiltration process allowed a final conductivity, for RhrBR46 and RhrBr46-Mg solutions, ranging from 114.6 to 127.5 µS∙cm^−1^ corresponding to about 6% [NaCl] equivalent. The high uronic acid content (88.12% and 73.20%) and the absence of neutral sugars (<0.1%) led us to consider RhrBR46 and RhrBR46-Mg as polyuronic acids. The polyuronic nature of these EPS was also supported by the high resistance of these fractions to acid hydrolysis due to the uronosydil linkages [40]. RhrBR46 and RhrBR46-Mg were partly resistant to H_2_SO_4_ since no hydrolysis occurred after various acid treatments, that is, 1 M H_2_SO_4_ at 100 °C from 30 min to 12 h or 2 M TFA 120 °C for 90 min. This resistance toward acid hydrolysis is a specific feature assigned to uronic acid-rich polysaccharides [12].

#### 3.2.2. Structural Features Analysis by FTIR and NMR

##### FTIR Spectroscopy

The presence of characteristic functional groups of polysaccharides can be partially identified using FTIR [41,42,43]. As RhrBR46 and RhrBR46-Mg were strongly suspected as polyuronic acids, the FTIR analysis was both carried out on the EPS and a known polyglucuronic acid (glucuronan SmPGU) originating from *S. meliloti* mutant M5N1CS strain [15,31], used as reference.

**Figure 2:**
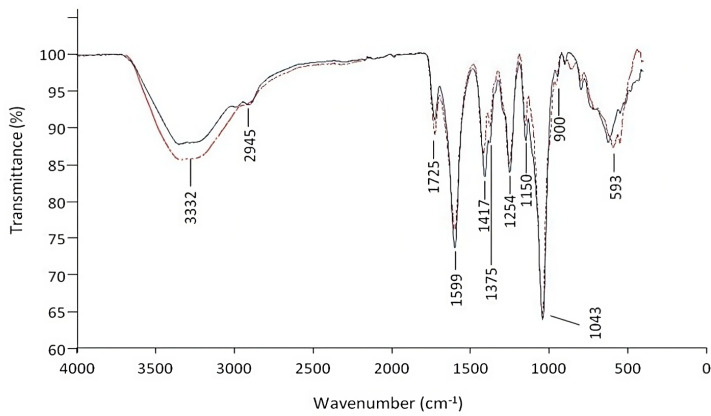
FTIR footprint spectra of RhrBR46 (black line) and RhrBR46-Mg (dark red dotted line). The wavenumbers of peaks are indicated.

The FTIR spectra of RhrBR46 and RhrBR46-Mg (Figure 2) showed, in the region of 3600–3000 cm^−1^ characteristic of (OH–) vibrations, a broad absorption band centered at 3332 cm^−1^ assigned to (OH–) stretching of polysaccharides and residual water (OH–) [43,44]. The absorption band at 2946 cm^−1^ was attributed to (CH–) asymmetric vibrations, classically observed in the region of 3000–2800 cm^−1^. In the region of 1800–1200 cm^−1^, related to the deformation vibrations of groups with local symmetry, the presence of the bands at 1599 cm^−1^ and 1417 cm^−1^, corresponding, respectively, to asymmetric and symmetric stretching modes of the planar COOH groups, confirmed the presence of uronic residues. The two bands, observed at 1725 cm^−1^ and 1250 cm^−1^ were assigned, respectively, to (C=O) stretching of ester groups and C–O–C antisymmetric stretching. The absorption band at 1725 cm^−1^ was indicative of acetate groups, demonstrating the acetylated feature of RhrBR46 and RhrBR46-Mg, whereas the band at 1250 cm^−1^ proved that the COOH functional group at *C*-6 could only take the carboxylate form [31]. The main band, observed at 1043 cm^−1^ and associated with (C–O) vibrations, is typically found in polysaccharides. In the anomeric region (950–700 cm^−1^), the observation of a small band at 900 cm^−1^, related to C1 anomeric conformation, allowed considering β-glycosidic linkages between residues of RhrBR46 and RhrBR46-Mg [45,46]. The FTIR spectrum of RhrBR46 was compared with those of the glucuronan from *S. meliloti* M5N1CS mutant strain used as a reference. The FTIR profile of glucuronan (Supplementary Figure S1) was shown consistent with previous studies [31] and close to the RhrBR46 spectrum.

Although FTIR analysis did not allow us to establish unambiguously the polysaccharide nature of RhrBR46 and RhrBR46-Mg, all these results might suggest these EPS as polyglucuronic acids (glucuronan).

##### NMR Profiles and Degree of Acetylation

^1^H and ^13^C NMR spectra of native and deacetylated RhRBR46 and RhRBR46-Mg are given in the Supplementary Figures S2–S4. The ^13^C and ^1^H NMR spectra of deacetylated RhrBR46 and RhrBR46-Mg revealed similar polymers having only five protons and six carbons of glucuronic acid were detected. Indeed, the ^1^H NMR spectrum of deacetylated RHRBR46 obtained after alkaline deacetylation of this polysaccharide has signals at 3.9, 4.18, 4.25, 4.42, and 5.11, respectively, attributed to H-2, H-3, H-4, H-5, and H-1 of glucuronic acid. In the ^13^C NMR signals at 175, 103, 82, 76, 74, and 72 ppm have been assigned to *C*-6, *C*-1, *C-*4, *C*-5, *C*-3, and *C*-2 of uronic acids β-(1,4) linked. The ^1^H of native polyuronides revealed a complex ring proton region between 2.4 and 2.6 ppm. These signals are characteristic of acetyl groups of an acetylated polysaccharide. Therefore, the assignments of native polysaccharides (RhrBR46 and RhrBR46-Mg) are given in Table 2 according to the literature [15,16,47], and assessed the previous statements about the structure of RhrBR46 and RhrBR46 as glucuronan with a main backbone composed of β-(1,4) linked glucuronic acid residues partially acetylated on 2 and/or 3 positions.

The degrees of substitution ($\bar{\mathrm{DS}}$) related to acetate groups (DA) were estimated both from FTIR and ^1^H NMR spectra from Table 2, using the equations given in Section 2.6.5. The results are shown in Table 3. The DA values obtained by FTIR ranged from 14 to 24.4% regardless of the calculation methods. The DA (%) of RhrBR46 and RhrBR46-Mg were close to 14–16% and 18–22%, respectively, indicating a direct impact of Mg^2+^ ions on the acetylation degree of the EPS. The DA of RhrBR46 appeared lower than that for the reference SmPGU estimated at 20–25%. It could be assigned to the variability of the culture conditions as previously observed [16,31]. The DAc (per disaccharide) was estimated to 0.67, 1.06, and 1.04, respectively, for RhrBR46, RhrBR46-Mg, and SmPGU.

The distributions of acetyl groups in RhrBR46 were identified and allocated as 19.8%, 59.5%, and 20.7%, respectively, for 2-*O*-Ac-, 3-*O*-Ac-, and 2,3-di-*O*-Ac-β-residues (Table 3). A similar pattern was obtained for SmPGU, indicating that RhrBR46 and SmPGU shared the same type of substitution groups, and only differed by the DA (%). Considering RhrBR46-Mg, a slight increase of 2,3-di-*O*-Ac-residues representation (29.4%) was observed compared to RhrBR46 (20.7%) as for the glucuronan of *S. meliloti* in presence of Mg^2+^ [17].

Magnesium ions seemed to have a direct impact on the DA of RhrBR46, without any knowledge of the action of Mg^2+^ ions on acetylation. One hypothesis would be to consider the effect of Mg^2+^ concentration on the enhancement of acetyltransferase activities, associated with the EPS biosynthesis, as it has been reported for the UDP-N-acetylglucosamine pyrophosphorylase/glucosamine-1-phosphate N-acetyltransferase (GlmU), a bifunctional enzyme catalyzing the synthesis of UDP-N-acetylglucosamine, an essential precursor of cell wall peptidoglycan and lipopolysaccharide synthesis in bacteria [48].

#### 3.2.3. Enzymatic Assays Using Glucuronan Lyase from *Trichoderma reseei* (TrGL)

**Figure 3:**
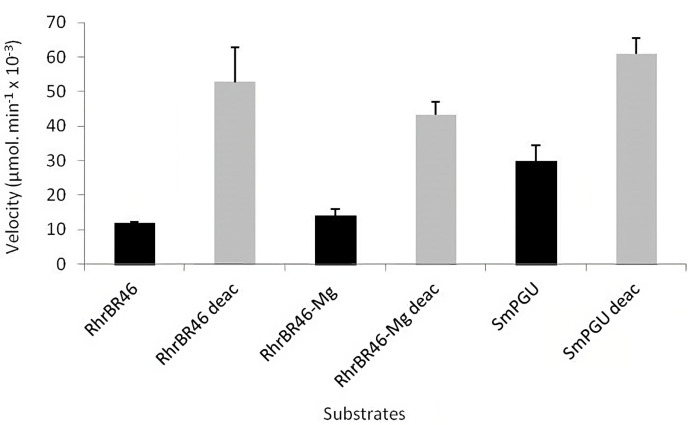
Analysis of the TrGL glucuronan lyase activity (from *Trichoderma reesei*) on RhrBR46, RhrBR46-Mg and SmPGU (reference) substrates and their deacetylated forms. All assays were carried out in triplicate (n = 3).

The analyses of the structural features were also made by testing its degradation (β-elimination) by the glucuronan lyase from *T. reseei*, described to be specifically active against β-(1,4)-glycosidic linkage of polyglucuronic substrates [49,50]. Deacetylated glucuronan is conventionally used to test TrGL activity as the enzyme was shown active specifically on this substrate. Deacetylated RhrBR46, RhrBR46-Mg and the reference SmPGU were also treated by 2 M KOH and the deacetylation was confirmed by ^1^H and ^13^C NMR (see Supplementary Figure S4). The activity of TrGL on the three native substrates and their deacetylated forms are presented in Figure 3.

RhrBR46 and RhrBR46-Mg were degraded (β-elimination) by TrGL indicating and confirming they are both polymers of β-(1,4)-polyglucuronic acids. The higher TrGL activities were observed using deacetylated polyuronides. This result confirmed previous ones revealing that the activity of the TrGL was inversely proportional to the substitution degrees of glucuronan by acetates [49]. In addition, the similar kinetics of degradation observed for the three deacetylated substrates (Figure 3) demonstrated on the one hand, that RhrBR46, RhrBR46-Mg and SmPGU had a similar backbone, and on the other hand, that the difference of TrGL activities observed between the three native forms (Figure 3) was related to the acetylation pattern.

Surprisingly, the TrGL activity was higher on SmPGU than RhrBR46, and similar between RhrBR46 and RhrBR46-Mg, whereas the FTIR and NMR analyses of DA showed a higher DA of RhrBR46 and RhrBR46-Mg compared, respectively, to SmPGU and RhrBR46. Considering SmPGU and RhrBR46, one hypothesis would be to consider a differential distribution of acetate groups along the polymer backbone, with a homogenous repartition for RhrBR46 and a disparity of distribution for SmPGU leading to acetylated-enriched regions on the polymer. As three non-acetylated residues of glucuronic acid (dp 3) are required as the main pattern for the endolyase action of TrGL [50], a homogenous distribution of acetylated residues might reduce the frequency of regions with a low abundance of non-acetylated residues, and consequently restrict the interaction or affinity of the enzyme to the polymer. The determination of enzymatic parameters of TrGL on both SmPGU and RhrBR46 (Table 4), seemed to support this hypothesis as demonstrated by a better affinity of TrGL for SmPGU (K_M_ 0.351 g∙L^−1^) compared to RhrBR46 (K_M_ 1.088 g∙L^−1^), resulting, thereby, in a 3.5 times higher efficiency (K_cat_/K_M_).

In the context of a homogeneous distribution of acetate groups on RhrBR46, the similar activity of TrGL observed for RhrBR46 and RhrBR46-Mg might suggest that the acetylation increased in presence of Mg^2+^, could be mainly directed to mono-acetylated (2-*O*- or 3-*O*-Ac) residues, generating consequently 2,3-di-*O*-Ac residues. This would result in a higher DAc and the increased rate of 2,3-di-*O*-Ac residues, which was consistent with that observed for RhrBR46-Mg (Table 3). Accordingly, the non-acetylated region’s representation of RhrBR46-Mg would not be affected by acetylation, which could explain the similar activity of TrGL for RhrBR46 and RhrBR46-Mg.

#### 3.2.4. Homogeneity and Molecular Weight (M_w_) Determination

The final assessment of the structural features was the determination of homogeneity and molecular mass of RhrBR46 and RhrBR46-Mg, carried out by HPSEC and SEC-MALS approaches. Spectra of elution profiles are shown in Supplementary Figure S3 and the results are summarized in Table 5.

Regarding HPSEC, two sets of columns (5000–3000 and 3000–2500) were used and molecular mass was determined using various pullulans as standards. The Polydispersity Index (Đ) ranging from 1.29 to 2.15 for RhrBR46 and RhrBR46-Mg, indicated a relatively homogeneous molar mass distribution for the two EPS. The average $\bar{M_{w}}$ of RhrBR46 and RhrBR46-Mg was shown to be quite similar, with an estimation close to 2 × 10^5^ Da with 5000–3000 columns and slightly lower (about 1.6 × 10^5^ Da) using 3000–2500 columns. This difference in $\bar{M_{w}}$ estimation was attributed to the limit of resolution/separation of each set of columns, generating an inaccuracy of molecular mass of the EPS, leading to a slight overestimation and underestimation of $\bar{M_{w}}$ with 5000–3000 and 3000–2500 columns, respectively. The $\bar{M_{w}}$ measured by HPSEC-MALS highlighted different molecular masses compared to HPSEC, with an estimation of 1.85 × 10^5^ Da and 4.9 × 10^4^ Da for RhrBR46 and RhrBR46-Mg, respectively (Table 4). The polydispersity index (Đ) (3.40 and 2.5) and the percentage of recovery (73% and 63%) confirmed, respectively, the homogeneity of molar mass distribution and the relative purity of the two EPS. The difference of $\bar{M_{w}}$ calculated by HPSEC and SEC-MALS, was from the methodologies. Estimating the $\bar{M_{w}}$ only by HPSEC, thanks to various pullulan standards, generated a bias due to conformational differences of the polymers in solution. Similar $\bar{M_{w}}$ were obtained for RhrBR46 and RhrBR46-Mg, whereas the $\bar{M_{w}}$ of RhrBR46-Mg was significantly lower by SEC-MALS. This difference can only be seen thanks to MALS and comes from the higher level of acetylation of RhrBR46-Mg, which increases the stiffness of the structure, thus leading to a drop in the hydrodynamic volume (see Supplementary Figure S5). The SEC-MALLS methodology, using the light scattering method, was, therefore, more adapted to give a real estimation of molecular mass, which was finally established to 1.85 × 10^5^ Da for RhrBR46 and 4.9 × 10^4^ Da RhrBR46-Mg.

The significant drop (~73%) of the $\bar{M_{w}}$ of RhrBR46 in enriched Mg^2+^ fermentation conditions, highlighted a direct impact of Mg^2+^ on the size of RhrBR46. These results were comparable to those obtained by Michaud et al. [17], who observed the reduction of SmPGU from 750,000 Da to 150,000 Da in presence of Mg^2+^. The identification of a glucuronan lyase [24,39], produced by *S. meliloti* during fermentation, has led the authors to consider its potential role in the preferential degradation of low-acetylated regions of the EPS, resulting in the M_w_ reduction and the enrichment in acetylated fractions. Currently, genes and chromosomal loci involved in the biosynthesis pathway of glucuronan remain unknown, whereas several gene clusters related to different exopolysaccharides biosynthesis have been characterized in various bacteria [51,52,53]. In *Pseudomonas*, the gene cluster involved in alginate biosynthesis pathway has genes encoding acetyltransferases acting on the acetylation of the polysaccharides [54,55], and an alginate lyase which has been demonstrated essential for the periplasmic translocation and secretion of EPS [56,57,58]. The characterization in *P. rosettiformans* of the genes cluster involved in glucuronan biosynthesis is a relevant way to better understand the pathway of production of glucuronan, and the potential roles of enzymes (acetylases and lyases) on the regulation of the size and the acetylation of RhrBR46.

## 4. Conclusions

In this study, we have identified a *Peteryoungia rosettiformans* strain, synthesizing a homopolymer of (1,4)-β-d-polyglucuronic acids (RhrBR46) related to glucuronan family. The structural features, determined by FTIR, NMR, and SEC-MALS allowed us to determine the molecular mass ($\bar{M_{w}}$) of 185,000 Da and the degree of acetylation (DA) reaching 14%, with a distribution of acetyl groups characterized at position 2-*O*-Ac (19.8%), 3-*O*-Ac (59.5%), and 2,3-di-*O*-Ac (20.7%) residues. The Mg^2+^ ions were proven to impact the structural features of the EPS by reducing its size by 73% and increasing of 10% its degree of acetylation (DA), without any antibacterial effect on the culture growth. The study of enzymatic degradability of RhrBR46 by the recombinant TrGL glucuronan lyase highlighted a lower affinity (K_M_) of the enzyme for RhrBR46 compared to SmPGU glucuronan, suggesting a differential distribution of acetyl group along the polymer between RhrBR46 and SmPGU. Finally, the strain *P. rosettiformans* constitutes the first non-mutant or recombinant producer of glucuronan (RhrBR46) isolated from the natural environment. The structural specificities of RhrBR46 might potentially lead to specific properties or biological activities that may constitute a real interest for novel applications.

## Figures and Tables

**Table 1:** Chemical compositions of RhrBR46 and RhrBR46-Mg.

EPS	Total Sugar (% *w*/*w*)	Neutral Sugar (% *w*/*w*)	Uronic Acids (% *w*/*w*)	Proteins (% *w*/*w*)	Phenolic Compounds (% *w*/*w*)	[NaCl] eq. * (% *w*/*w*)	Conductivity (µS∙cm^−1^)
RhrBR46	78.22 ± 1.72	<0.1	88.15 ± 0.75	0.38 ± 0.03	ND	5.99	127.5
RhrBR46-Mg	65.17 ± 1.10	<0.1	73.20 ± 0.42	0.17 ± 0.004	ND	5.40	114.6

* [NaCl] equivalent; ND: Not Detected.

**Table 2:** Assignment of chemical-shit values of RhrBR46 and RhrBR46-Mg.

Compounds	δ (ppm)										
^1^H	A	B	C	D	E	F	G	H	I	J	K
RhrBR46	5.38	5.18	5.08	4.99	4.92	4.90	4.82	4.30–3.62	2.54	2.48	2.40
RhrBR46-Mg	5.57	5.44	5.24	5.15	5.06	4.99	4.89	4.43–3.7	2.60	2.53	2.47
^13^C	*C*-1		*C*-2		*C*-3		*C*-4		*C*-5		*C*-6
RhrBR46	102.86		73.33		73.56		81.47		75.95		174.05
RhrBR46-Mg	102.76		73.32		73.53		81.19		75.93		173.77
A: H3 of a 2,3-di-*O*-acetylated glucuronic acid.											
B: H3 of a 3-*O*-acetylated glucuronic acid.											
C: H1 + H2 of a 2,3-di-*O*-acetylated glucuronic acid.											
D: H1 + H2 of a 2-*O*-acetylated glucuronic acid. E: H1 of a 3-*O*-acetylated glucuronic acid.											
F: H1 of a non-acetylated glucuronic acid inside a non-acetylated group.											
G: H1 of a non-acetylated glucuronic acid before or after an acetylated residue.											
H: Signals attributed to H2, H3, H4 and H5 from the repetitive unit.											
I: Protons of an acetyl group in *C*-2 position of a 2-*O*-acetylated glucuronic acid. J: Protons of an acetyl group in *C*-3 position of a 3-*O*-acetylated glucuronic acid and in *C*-2 position of a 2,3-di-*O*-acetylated glucuronic acid. K: Protons of an acetyl group in *C*-3 position of a 2,3-di-*O*-acetylated glucuronic acid.											

**Table 3:** Analysis of the acetylation profile of RhRBR46 and RhrBR46-Mg.

	FTIR			NMR				
Samples	DA (%) (A_1725_/A_1045_)	DA (%) (A_1725_/A_1598_)	DA (%) (A_1725_/A_1045+1598_)	DAc per Residue ^(^*^)^	DAc per Disaccharide ^(^**^)^	Distribution (%)		
						2-*O*-Ac	3-*O*-Ac	2,3-di-*O*-Ac
RhrBR46	14.05	16.3	14.0	0.37	0.67	19.8	59.5	20.7
RhrBR46-Mg	17.85	22.2	18.2	0.47	1.06	17.5	53.1	29.4
SmPGU	21.2	24.4	19.6	0.48	1.04	20	55.8	24.2

(*) DAc obtained from Equation 7 (see Section 2); (**) DAc obtained from Equation (8) (see Section 2).

**Table 4:** Determination of enzymatic parameters of the TrGL enzyme on RhrBR46 and SmPGU. The values correspond to the means of three experimental repetitions (n = 3).

Samples	V_M_ (µmol∙min^−1^)	K_M_ (g∙L^−1^)	K_cat_ (g∙L^−1^)	K_cat_/K_M_ (min^−1^∙g^−1^∙L)
RhrBR46	0.043 ± 0.0054	1.088 ± 0.0358	0.627	0.577
SmPGU	0.049 ± 0.0114	0.351 ± 0.0255	0.715	2.039

**Table 5:** Determination of $\bar{M_{w}}$ of RhrBR46 and RhrBR46-Mg.

EPS	Separation	Set of Columns	Recovery %	M_n_ (g∙mol^−1^)	Deviation	M_w_ (g∙mol^−1^)	Deviation	(Ð)
RhrBR46	HPSEC	5000–3000	-	1.41 × 10^5^	0.10 × 10^5^	2.01 × 10^5^	7 × 10^3^	1.42
		3000–2500	-	1.19 × 10^5^	5.63 × 10^2^	1.54 × 10^5^	1.65 × 10^3^	1.29
	SEC-MALS	804-806 HQ	73	0.55 × 10^5^	-	1.85 × 10^5^	-	3.40
RhrBR46-Mg	HPSEC	5000–3000	-	0.93 × 10^5^	0.11 × 10^5^	2 × 10^5^	0.13 × 10^5^	2.15
		3000–2500	-	1.16 × 10^5^	1.28 × 10^3^	1.67 × 10^5^	3.14 × 10^3^	1.44
	SEC-MALS	804-806 HQ	63	0.2 × 10^5^	-	4.9 × 10^4^	-	2.5

## Data Availability

Not applicable.

## Supplementary Materials

The following supporting information can be downloaded at: https://www.mdpi.com/article/10.3390/polym15092177/s1, Figure S1: FTIR footprint spectra of RhrBR46 (black line) and the reference glucuronan SmPGU (red dotted line). The wavenumbers of peaks are indicated (in brackets for SmPGU); Figure S2: ^1^H and ^13^C NMR spectra of RhrBR46. The proton assignments are given as follows: H3 of a 2,3-di-*O*-acetylated residue (A), H3 of a 3-*O*-acetylated residue (B), H1 + H2 of a 2,3-di-*O*-acetylated residue (C), H1 + H2 of a 2-*O*-acetylated residue (D), H1 of a 3-*O*-acetylated residue (E), H1 of a non-acetylated residue inside a non-acetylated group (F), H1 of a non-acetylated residue before or after an acetylated residue (G), signals attributed to H2, H3, H4, and H5 from the repetitive unit (H), protons of an acetyl group in *C*-2 position of a 2-*O*-acetylated residue (I), protons of an acetyl group in *C*-3 position of a 3-*O*-acetylated residue and in *C*-2 position of a 2,3-di-*O*-acetylated residue (J), and protons of an acetyl group in *C*-3 position of a 2,3-di-*O*-acetylated residue (K); Figure S3: ^1^H and 1^3^C NMR spectra of RhrBR46-Mg. The proton assignments are given as follows: H3 of a 2,3-di-*O*-acetylated residue (A), H3 of a 3-*O*-acetylated residue (B), H1 + H2 of a 2,3-di-*O*-acetylated residue (C), H1 + H2 of a 2-*O*-acetylated residue (D), H1 of a 3-*O*-acetylated residue (E), H1 of a non-acetylated residue inside a non-acetylated group (F), H1 of a non-acetylated residue before or after an acetylated residue (G), Signals attributed to H2, H3, H4, and H5 from the repetitive unit (H), protons of an acetyl group in *C*-2 position of a 2-*O*-acetylated residue (I), protons of an acetyl group in *C*-3 position of a 3-*O*-acetylated residue and in *C*-2 position of a 2,3-di-*O*-acetylated residue (J), and protons of an acetyl group in *C*-3 position of a 2,3-di-*O*-acetylated residue (K); Figure S4: ^1^H and ^13^C spectra NMR of deacetylated RhrBR46, RhrBR46-Mg and the reference SmPGU. The proton assignments are given as following: Signals attributed to H2, H3, H4, and H5 from the repetitive unit (H), protons of an acetyl group in *C*-2 position of a 2-*O*-acetylated residue (I), protons of an acetyl group in *C*-3 position of a 3-*O*-acetylated residue and in *C*-2 position of a 2,3-di-*O*-acetylated residue (J), and protons of an acetyl group in *C*-3 position of a 2,3-di-*O*-acetylated residue (K); Figure S5: Elution profiles obtained by SEC/MALS/DRI from refractive index (full lines) and LS (dotted lines) of RhrBR46 (black) and RhrBR46-Mg (red) together with molar masses distribution in LiNO_3_ 0.1 mol∙L^−1^.
