# Supplementary material for: Description of the Wild Strain Rhizobium rosettiformans DSM26376, Reclassified under Peteryoungia rosettiformans comb.nov., for Producing Glucuronan
Source: Polymers (Basel). 2023 May 3;15(9):2177. doi: 10.3390/polym15092177 (PMC10180729; doi:10.3390/polym15092177)
Supplement: Supplementary file 1 [file polymers-15-02177-s001.zip › polymers-2244243-supplementary.pdf]

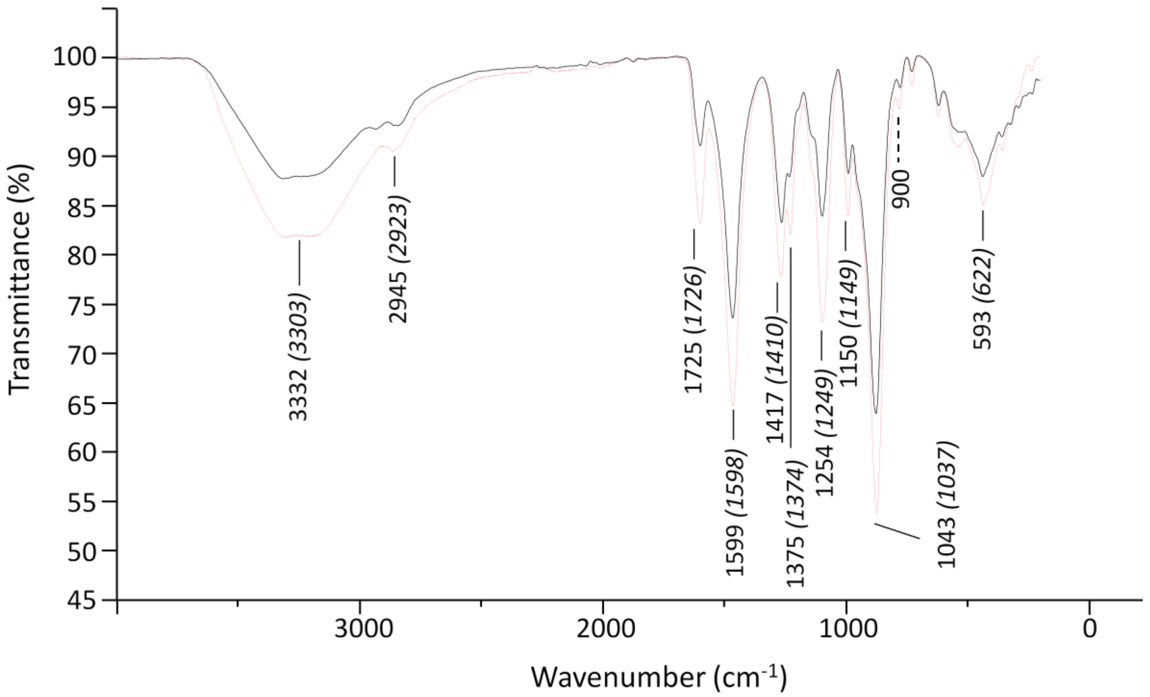

Figure S1. FTIR footprint spectra of RhrBR46 (black line) and the reference glucuronan SmPGU (red dotted line). The wavenumbers of peaks are indicated (in brackets for SmPGU).

^{1}H RhrBR46

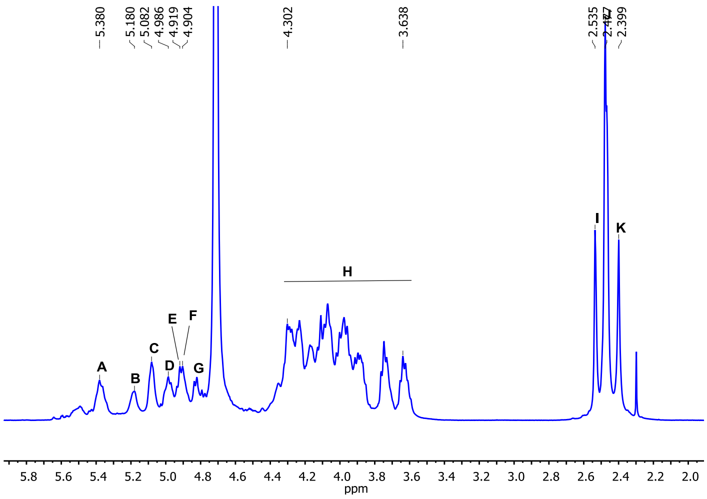

^{13}C RhrBR46

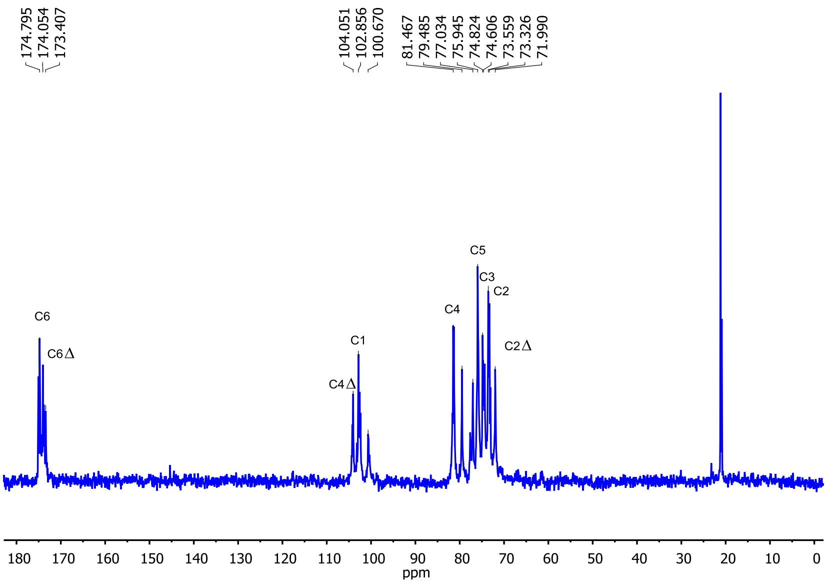

Figure S2. ^{1}H and 1^{3}C RMN spectra of RhrBR46. The proton assignments are given as following: : H3 of a 2,3-di-*O*-acetylated residue (A), H3 of a 3-*O*-acetylated residue (B), H1+H2 of a 2,3-di-*O*-acetylated residue (C), H1+H2 of a 2-*O*-acetylated residue (D), H1 of a 3-*O*-acetylated residue (E), H1 of a non-acetylated residue inside a non-acetylated group (F), H1 of a non-acetylated residue before or after an acetylated residue (G), Signals attributed to H2, H3, H4 and H5 from the repetitive unit (H), Protons of an acetyl group in *C*-2 position of a 2-*O*-acetylated residue (I), Protons of an acetyl group in *C*-3 position of a 3-*O*-acetylated residue and in *C*-2 position of a 2,3-di-*O*-acetylated residue (J) and Protons of an acetyl group in *C*-3 position of a 2,3-di-*O*-acetylated residue (K).

^{1}H RhrBR46-Mg

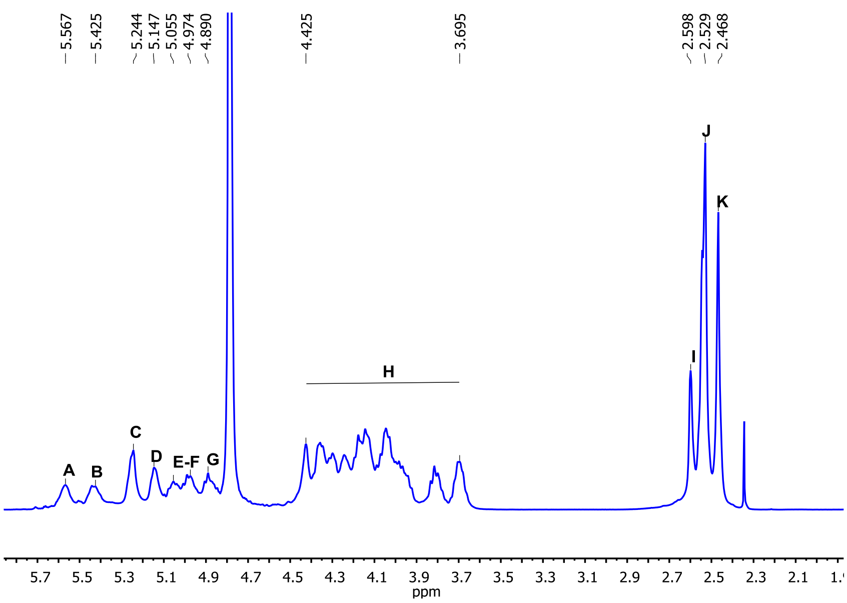

^{13}C RhrBR46-Mg

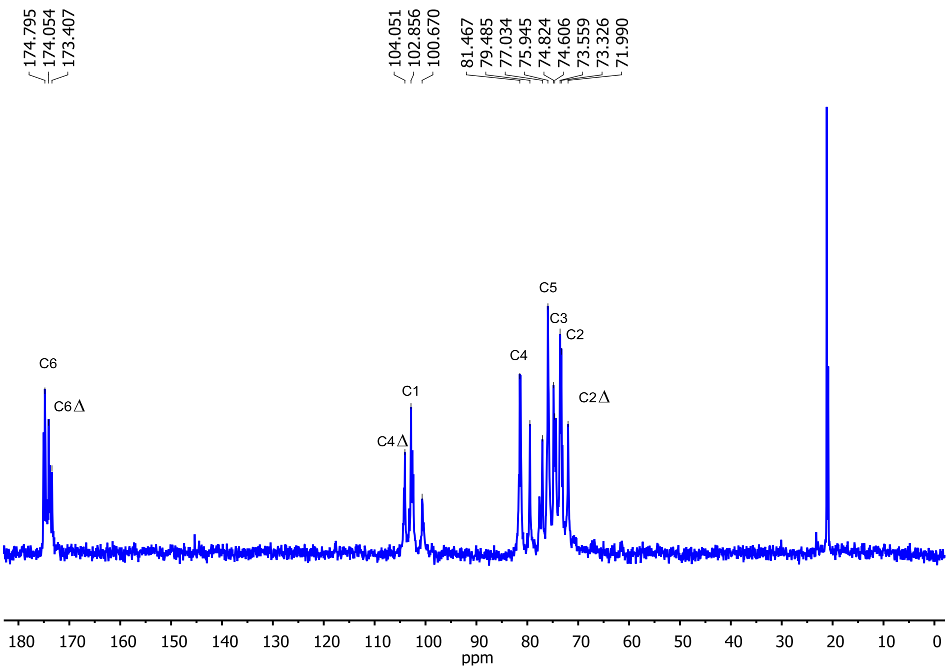

Figure S3. ^{1}H and ^{13}C RMN spectra of RhrBR46-Mg. The proton assignments are given as following: : H3 of a 2,3-di-*O*-acetylated residue (A), H3 of a 3-*O*-acetylated residue (B), H1+H2 of a 2,3-di-*O*-acetylated residue (C), H1+H2 of a 2-*O*-acetylated residue (D), H1 of a 3-*O*-acetylated residue (E), H1 of a non-acetylated residue inside a non-acetylated group (F), H1 of a non-acetylated residue before or after an acetylated residue (G), Signals attributed to H2, H3, H4 and H5 from the repetitive unit (H), Protons of an acetyl group in *C*-2 position of a 2-*O*-acetylated residue (I), Protons of an acetyl group in *C*-3 position of a 3-*O*-acetylated residue and in *C*-2 position of a 2,3-di-*O*-acetylated residue (J) and Protons of an acetyl group in *C*-3 position of a 2,3-di-*O*-acetylated residue (K).

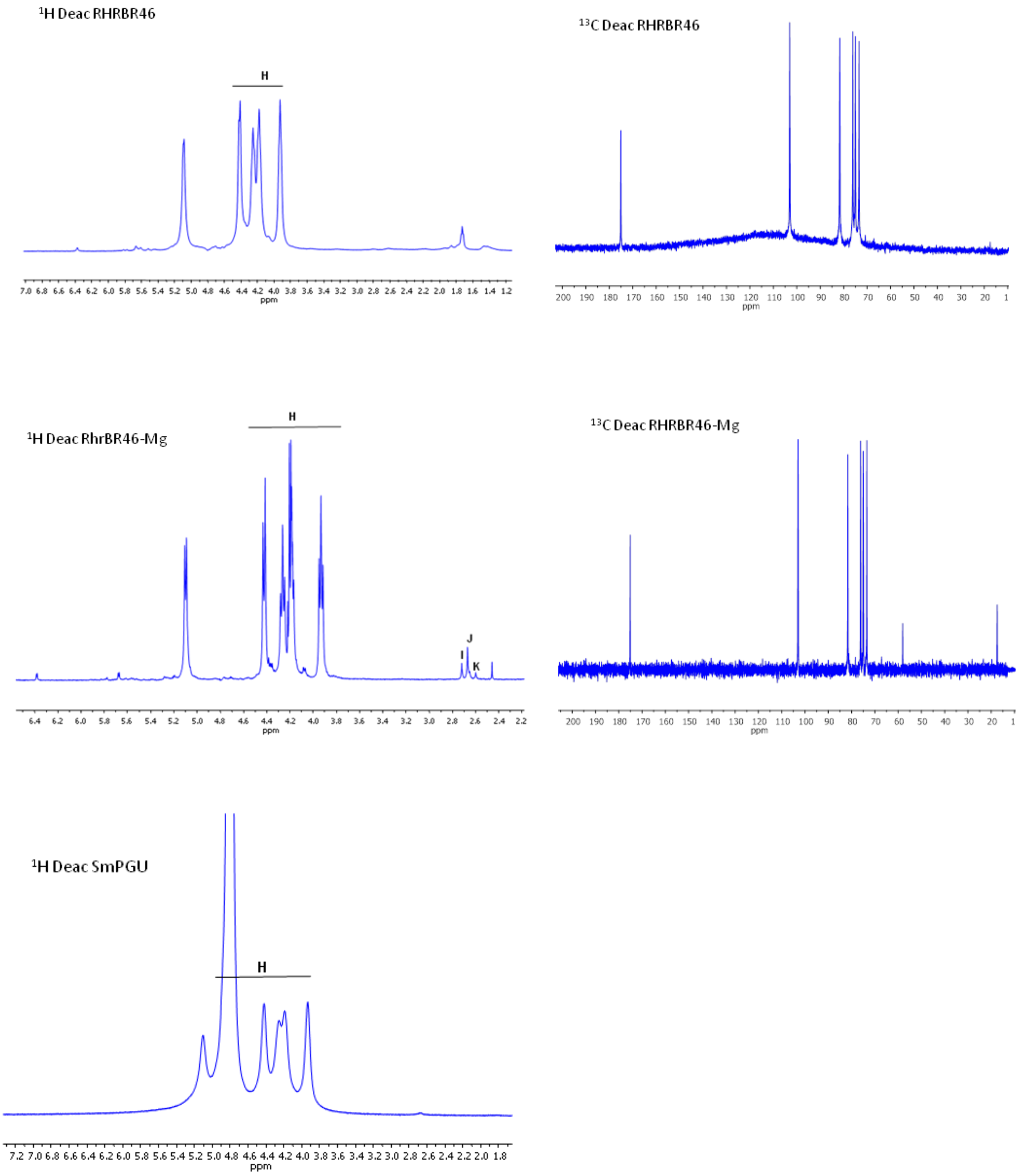

Figure S4. ^{1}H RMN and ^{13}C spectra of deacetylated RhrBR46, RhrBR46-Mg and the reference SmPGU. The proton assignments are given as following: : signals attributed to H2, H3, H4 and H5 from the repetitive unit (H), Protons of an acetyl group in *C*-2 position of a 2-*O*-acetylated residue (I), Protons of an acetyl group in *C*-3 position of a 3-*O*-acetylated residue and in *C*-2 position of a 2,3-di-*O*-acetylated residue (J) and Protons of an acetyl group in *C*-3 position of a 2,3-di-*O*-acetylated residue (K).

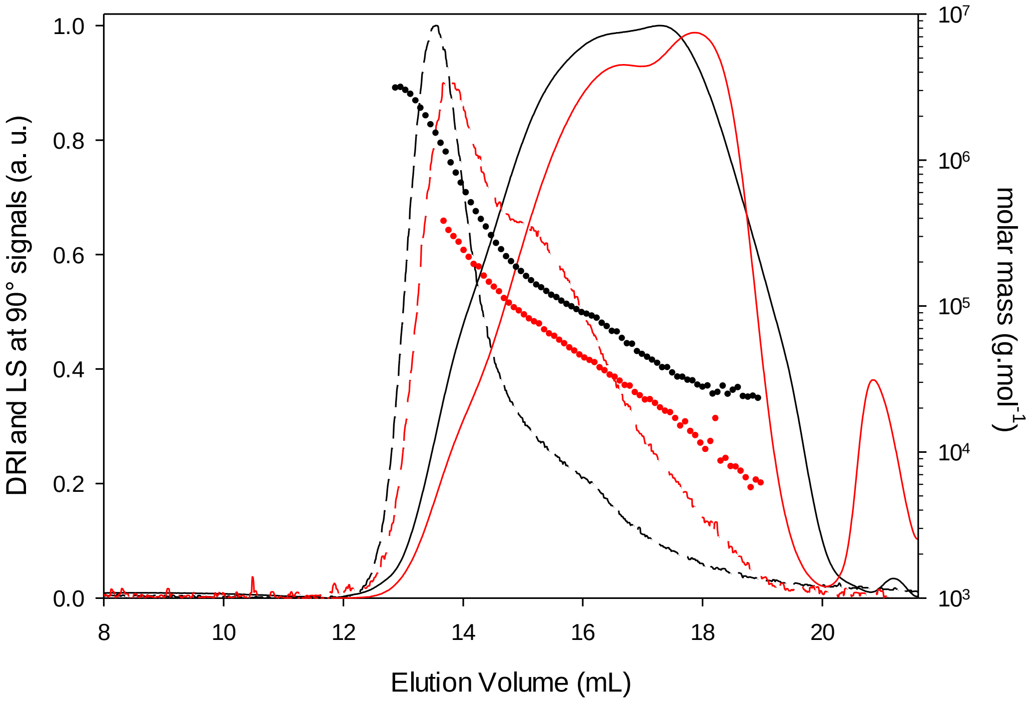

Figure S5. Elution profiles obtained by SEC/MALS/DRI from refractive index (full lines) and LS (dotted lines) of RhrBR46 (black) and RhrBR46-Mg (red) together with molar masses distribution in $LiNO_3$ 0.1 mol.L^{-1}.
